# ATRX is a predictive marker for endocrinotherapy and chemotherapy resistance in HER2-/HR+ breast cancer through the regulation of the AR, GLI3 and GATA2 transcriptional network

**DOI:** 10.18632/aging.205327

**Published:** 2023-12-20

**Authors:** Hongyan Qian, Rui Ji, Cheng Shen, Yinze Wei, Chenyi Sheng, Qichao Ni, Jing Pan, Yifan Chi, Huan You, Ying Miao, Minxin Shi, Xianghua Huang, Aiguo Shen

**Affiliations:** 1 Cancer Research Center Nantong, Nantong Tumor Hospital, Affiliated Tumor Hospital of Nantong University, Nantong 226361, China; 2 Department of Gynecology Oncology, Nantong Tumor Hospital, Affiliated Tumor Hospital of Nantong University, Nantong 226361, China; 3 Department of Computer Science and Engineering, Tandon School of Engineering, New York University, Brooklyn, NY 11201, USA; 4 Department of Surgery, Affiliated Tumor Hospital of Nantong University, Nantong 226361, China; 5 Department of Pathology, Affiliated Tumor Hospital of Nantong University, Nantong 226361, China; 6 Department of Breast Surgery, Affiliated Hospital of Nantong University, Nantong 226001, China; 7 School of Medicine, Nantong University, Nantong 226001, China

**Keywords:** ATRX, drug resistance, biomarker, breast cancer, transcription factor

## Abstract

Drug resistance in breast cancer (BC) is a clinical challenge. Exploring the mechanism and identifying a precise predictive biomarker for the drug resistance in BC is critical. Three first-line drug (paclitaxel, doxorubicin and tamoxifen) resistance datasets in BC from GEO were merged to obtain 1,461 differentially expressed genes for weighted correlation network analysis, resulting in identifying ATRX as the hub gene. ATRX is a chromatin remodelling protein, therefore, ATRX-associated transcription factors were explored, thereby identifying the network of AR, GLI3 and GATA2. GO and KEGG analyses revealed immunity, transcriptional regulation and endocrinotherapy/chemotherapy resistance were enriched. Moreover, CIBERSORT revealed immunity regulation was inhibited in the resistance group. ssGSEA showed a significantly lower immune status in the ATRX-Low group compared to the ATRX-High group. Furthermore, the peaks of H3K9me3 ChIP-seq on the four genes were higher in normal tissues than in BC tissues. Notably, the frequency of ATRX mutation was higher than BRCA in BC. Moreover, depressed ATRX revealed worse overall survival and disease-free survival in the human epidermal growth factor receptor 2 (HER2)-/hormone receptor (HR)+ BC. Additionally, depressed ATRX predicted poor results for patients who underwent endocrinotherapy or chemotherapy in the HER2-/HR+ BC subgroup. A nomogram based on ATRX, TILs and ER exhibited a significantly accurate survival prediction ability. Importantly, overexpression of ATRX significantly inhibited the IC_50_ of the three first-line drugs on MCF-7 cell. Thus, ATRX is an efficient predictive biomarker for endocrinotherapy and chemotherapy resistance in HER2-/HR+ BC and acts by suppressing the AR, GLI3 and GATA2 transcriptional network.

## INTRODUCTION

Breast cancer (BC) is the most commonly diagnosed malignancy and the fifth leading cause of cancer-related mortality worldwide, with an estimated 2.3 million new cases and 685,000 deaths in 2020 [1]. BC is characterized by four subtypes based on their expressions in estrogen (ER), progesterone (PR), and human epidermal growth factor receptor 2 (HER2). Among these subtypes, HER2-/ hormone receptor-positive (HR+) (i.e., ER and/or PR-positive) BC is the most common and least invasive one, accounting for 60%–65% of BC in China and 70% in Europe and America [2, 3]. Nevertheless, the prevention of late recurrence in this subtype, which occurs in 40% of patients after more than 10 years of diagnosis, is a major challenge [4]. Furthermore, approximately 30% of this subtype of patients can develop drug resistance (first-line drugs, such as paclitaxel (PTX), doxorubicin (DOX), tamoxifen (TMX) etc.) and metastasis [5]. Thus, the identification of a predictive biomarker for the first-line drugs resistance is particularly significant for patients with the HER2-/HR+ BC subtype.

ATP-dependent helicase ATRX was first reported in alpha thalassemia/mental retardation X-linked diseases in 1995 [6], but its role in tumours remained unexplored until the last decade. ATRX belongs to the switch/sucrose nonfermentable (SWI/SNF) family of chromatin remodelling proteins. It is located in the nucleus and regulates chromatin association. Studies have reported that ATRX mutation occurs in some mesenchymal tumours, such as glioma [7], pancreatic neuroendocrine tumours [8], paraganglioma [9] and pleomorphic sarcomas [10]. ATRX mutation leads to decreased ATRX protein expression and results in tumour genome instability and higher tumour mutation, making it a useful independent predictor of poor overall survival (OS) or disease-free survival (DFS) for the above tumours. To the best of our knowledge, there exist only a few reports on ATRX in epithelial tumours, such as gastric cancer [11], cervical carcinoma [12], hepatocarcinoma [13] and non-small cell lung cancer (NSCLC) [14]. ATRX mutation sensitises NSCLC to immune checkpoint inhibitors (ICIs), thereby highlighting the potential of ATRX as a promising biomarker for ICIs [14]. Additionally, survival analysis on cervical carcinoma suggested that the loss-of-function of ATRX was associated with a better prognosis in patients treated with chemo-radiation [12]. However, to date, the potential role of ATRX in BC, especially its role in the HER2-/HR+ subtype, remains inadequately explored [15].

Dysregulated transcription factors (TF) mediate aberrant gene expression, which is the hallmark properties of drug resistance in tumours [16]. Androgen receptor (AR), as a ligand-dependent nuclear TF and a steroid nuclear receptor, is frequently expressed in BC and has long been considered an attractive therapeutic target. It has been reported that TMX-resistant BC owned higher levels of AR than the corresponding-sensitive one [17]. GLI3 is a zinc finger TF, which has been found that GLI3 and AR are mutually dependent for the growth and migration of BC cells [18]. Nevertheless, whether the interaction of GLI3 and AR mediates drug resistance in BC has not been reported yet. GATA2 not only blocks AR-induced PTEN expression by preventing AR nuclear translocation, but also directly represses PTEN transcription independent of AR to promote BC cell growth [19]. Moreover, in prostate cancer, GATA2 has been well-characterized as a critical pioneer TF for AR, whereas GATA2 can mediate PTX resistance by AR-independent regulation of IGF2 [20]. All these studies remind that AR, GLI3 and GATA2 exist complex regulation in tumours. Whether they can mediate the resistance of the first-line drugs in BC is still unclear.

Variety TFs can affect chromatin pioneer functions through dynamic interaction with ATP-dependent chromatin remodelling proteins [21]. However, the significance of the regulation between ATRX and the above three TFs in the resistance of PTX, DOX or TMX is still unclear. In this study, we combined bioinformatics, retrospective study and cell experiment to investigate the potential predictive value of ATRX in first-line drugs resistance of HER2-/HR+ BC patients and tried to explore the mechanism preliminary so as to provide therapeutic strategy precisely for clinicians.

## RESULTS

### Function and pathway enrichment analysis of DEGs for drug resistance in BC

**Figure 1:**
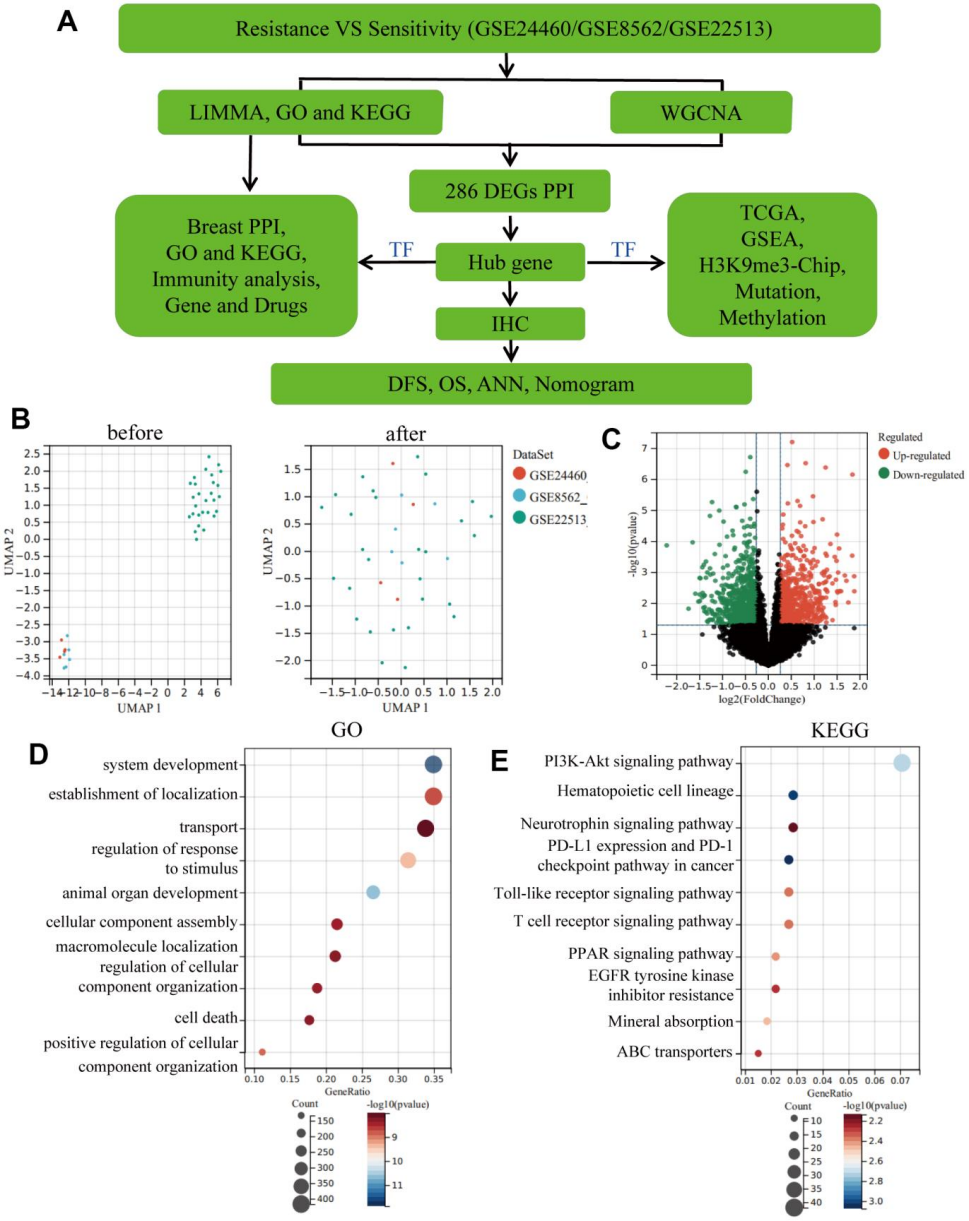
**Eliminating batch effect between different sequencing platforms.** (**A**) Workflow of this study. (**B**) Eliminating the batch effect of GSE24460, GSE862 and GSE22513, and combing the three datasets into a singular dataset, named GSE1. (**C**) Volcano plots of DEGs in GSE1. (**D**) TOP10 BP enrichment analysis of DEGs in GSE1. (**E**) KEGG enrichment of the TOP10 terms in GSE1.

The workflow of this study is shown in Figure 1A. To investigate the differentially expressed genes (DEGs) involved in the drug resistance of BC, we downloaded and merged three relevant GEO datasets to obtain a GSE1 dataset. Moreover, on eliminating the batch effect between different sequencing platforms, a total of 1,461 DEGs were screened, wherein 818 DEGs were downregulated and 650 DEGs were upregulated (Figure 1B, 1C). Gene Ontology (GO) and Kyoto Encyclopaedia of Genes and Genomes (KEGG) analysis on DEGs (Figure 1D, 1E) revealed that the TOP10 biological process (BP) of GO was focused on transport, regulation of response to stimulus and cellular component assembly. KEGG revealed the enrichment of immune-related signalling pathways like PD-L1 expression and PD-1 checkpoint pathway in cancer, Toll-like receptor signalling pathway and T cell receptor signalling pathway. Moreover, the PI3K-Akt signalling pathway, PPAR signalling pathway and ABC transporters, which are known as drug resistance signalling pathways, were also enriched, thereby further validating our results.

### Screening of hub genes using WGCNA and PPI

**Figure 2:**
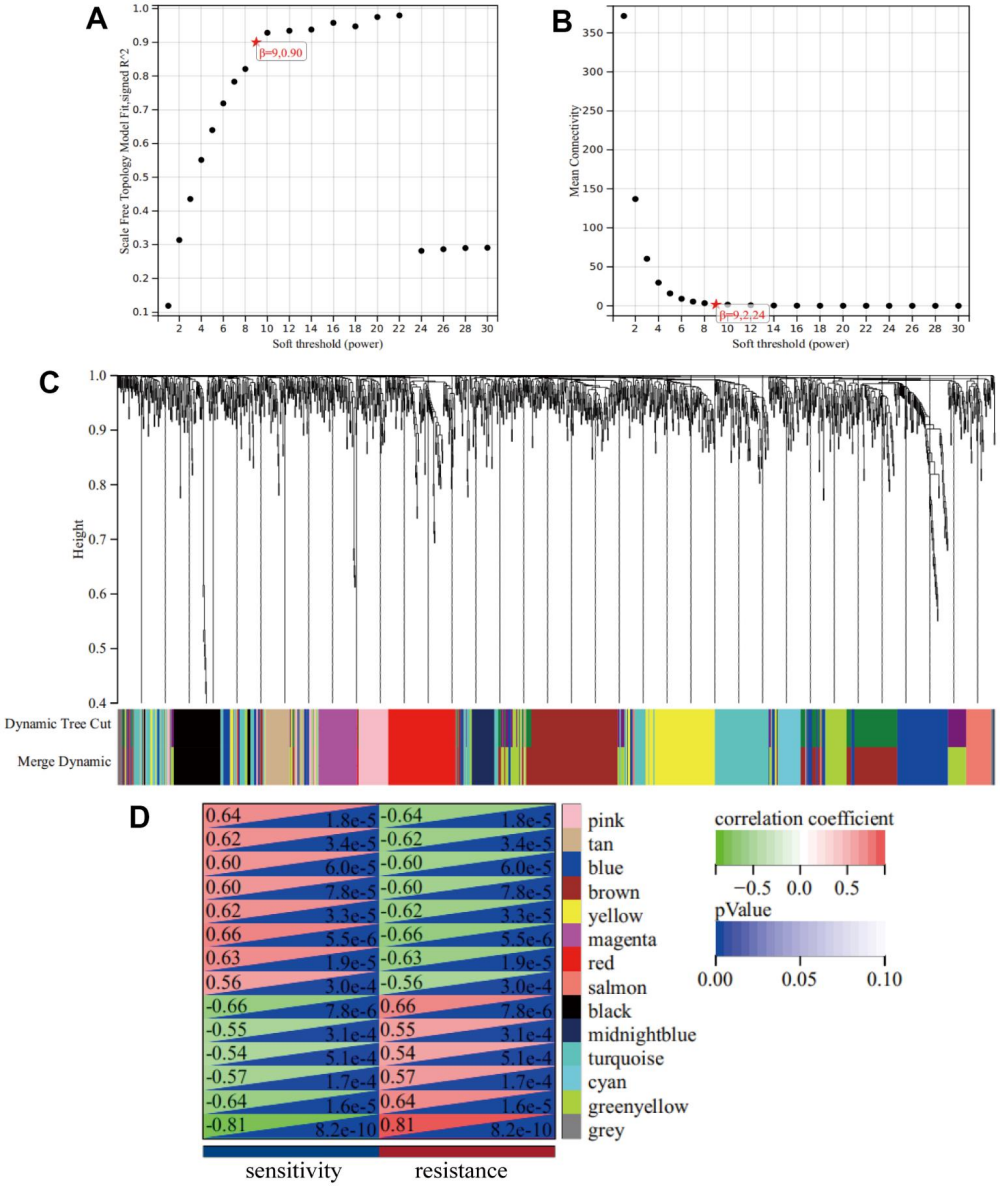
**WGCNA.** (**A**) A Scale-free fit index as a function of soft-thresholding power. (**B**) The mean connectivity is a function of soft-thresholding powers. (**C**) Highly interconnected groups of genes were clustered and modules are represented by distinct colours in the horizontal bar. (**D**) Heatmap showed the correlations of module eigengenes with clinical traits. The numbers in each cell represent the correlation coefficients and P-value between clinical trait and module eigengenes.

**Figure 3:**
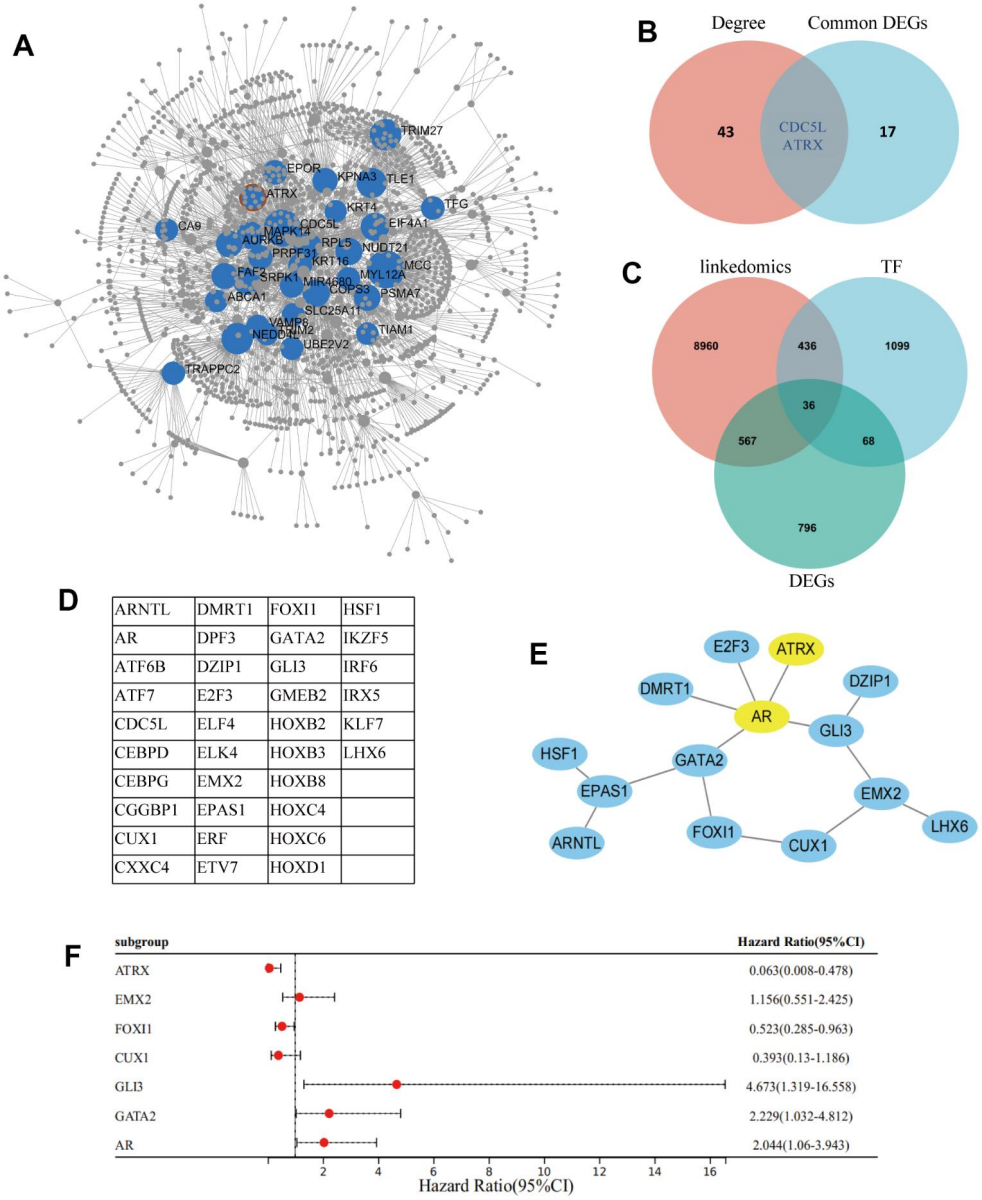
**Screening of hub gene.** (**A**) Network Analyst Database for breast mammary tissue PPI analysis on 286 DEGs, which were filtered by Degree ≥ 15. (**B**) Venn diagram reveals the hub genes. (**C**) Venn diagram detected 36 common DEGs of ATRX-associated, TFs and DEGs in GSE1. (**D**) The name of the 36 common DEGs. (**E**) STRING and Cytoscape analysis of the common DEGs. (**F**) Logistic regression analysis of genes related to drug resistance.

A cluster dendrogram of 1,461 DEGs was constructed using WGCNA based on the criteria of soft-threshold β = 9 and scale-free R^2^ = 0.9 (Figure 2A, 2B). Subsequently, 14 gene modules were identified in the hierarchical clustering, based on a merge cut height of 0.25 and a minimum module size of 30 (Figure 2C). Among them, the brown module (correlation coefficient = -0.60, P = 7.88×10^-5^) exhibited a significantly strong negative correlation with drug resistance. Therefore, the brown module was selected for further analysis as it contained 286 DEGs, which was the highest number of genes in a module, despite not having the strongest correlation with drug resistance (Figure 2D and Supplementary Table 2). Furthermore, 286 DEGs were selected for breast mammary tissue protein-protein interaction networks (PPI) analysis on a Network Analyst Database and 45 DEGs were identified using a Degree filter ≥ 15 (Figure 3A). Based on the intersection of DEGs from the three GEO datasets, we further identified 17 common DEGs that were downregulated and three common DEGs that were upregulated (Supplementary Figure 1A, 1B). Furthermore, ATRX and CDC5L were identified as hub genes using a Venn diagram of 45 DEGs from the brown module and 20 common DEGs (Figure 3B).

### Exploring the role of ATRX in the drug resistance of BC

ATRX, a chromatin remodelling element, plays an important role in transcriptional regulation. Hence, an intersection analysis of ATRX-related genes in BC from TCGA was performed using the LinkedOmics database, Human TFs and DEGs in GSE1, consequently identifying 36 common DEGs (Figure 3C, 3D). PPI analysis highlighted that ATRX combined with AR, GLI3, EMX2, CUX1, FOXI1 and GATA2 formed a regulation circle (Figure 3E). Cox univariate analysis also revealed that, apart from EMX2 and CUX1, ATRX (Hazard Ratio (HR) = 0.063, 95% confidence interval (CI): 0.008-0.478) and FOXI1 (HR = 0.523, 95% CI: 0.285-0.963) showed a negative correlation with drug resistance, while AR (HR = 2.044, 95% CI:1.06-3.943), GLI3 (HR = 4.673, 95% CI:1.319-16.558) and GATA2 (HR = 2.229, 95% CI:1.032-4.812) showed a positive correlation with drug resistance (Figure 3F).

**Figure 4:**
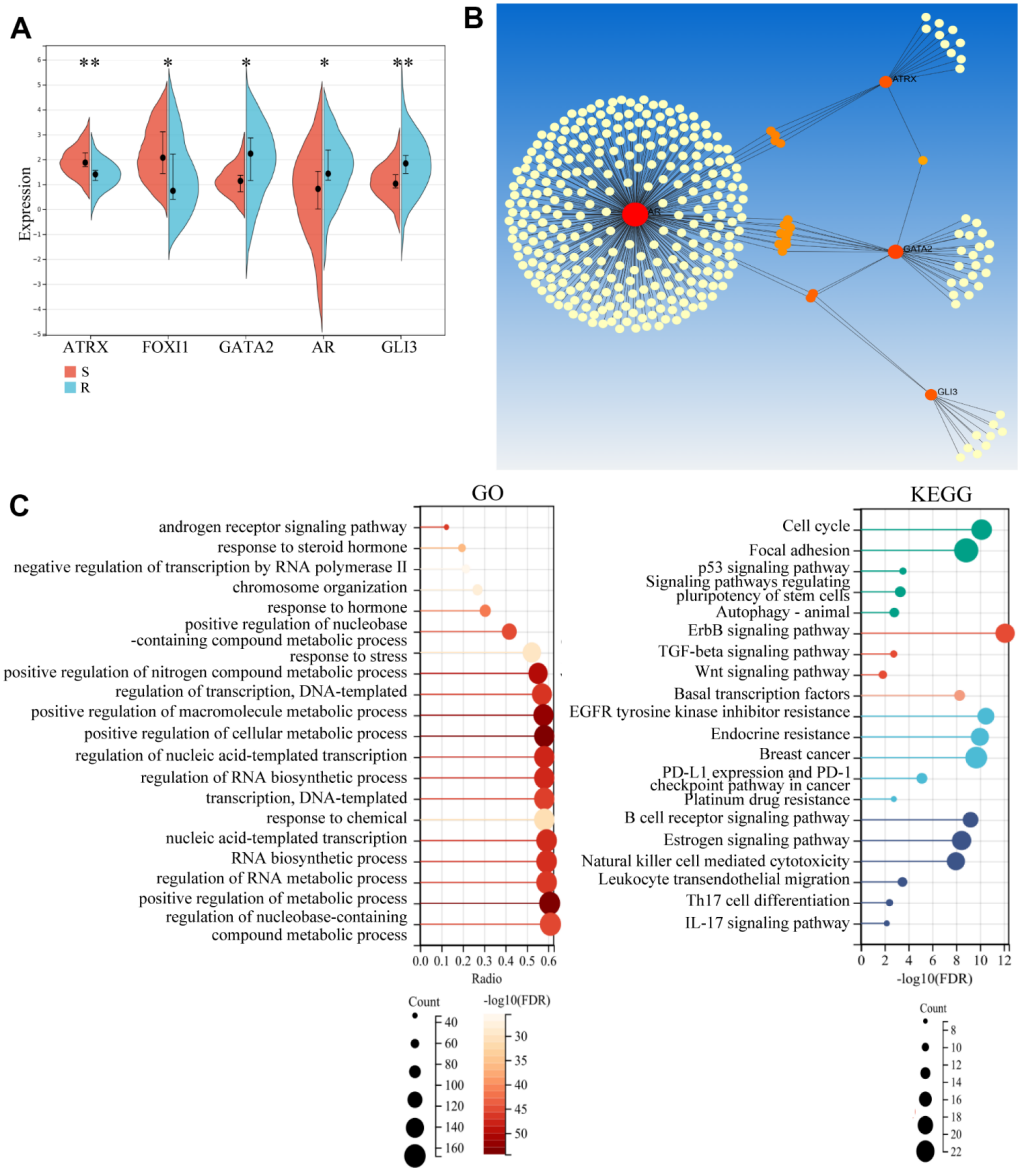
**Potential role of the hub gene and associated TFs.** (**A**) The expression of the five important genes in GSE1. (**B**) Network Analyst Database for PPI analysis on the five genes. (**C**) GO and KEGG analysis of the four genes using the Network Analyst Database.

To further explore the role of these genes, we first detected their expression. The resistant samples revealed a reduction in ATRX and FOXI1 expression and an increase in AR, GLI3 and GATA2 expression (Figure 4A), which was consistent with the data of BC from TCGA. Notably, most of them had significant differences in both DFS and OS analysis (Supplementary Figure 2). Following this, Network Analyst Database analysed these genes in breast mammary tissue PPI, revealing a regulation network composed of ATRX, AR, GLI3 and GATA2 (Figure 4B). Furthermore, GO analysis for the network showed that the AR signalling pathway, negative regulation of transcription by RNA polymerase II and chromosome organization were enriched. KEGG analysis also revealed that the PI3K-Akt signalling pathway, Basal transcription factors, Endocrine resistance and Platinum drug resistance were enriched. Moreover, immunity-regulation signalling pathways were particularly enriched: such as the B cell receptor signalling pathway, Natural killer cell-mediated cytotoxicity and T cell receptor signalling pathway (Figure 4C).

**Figure 5:**
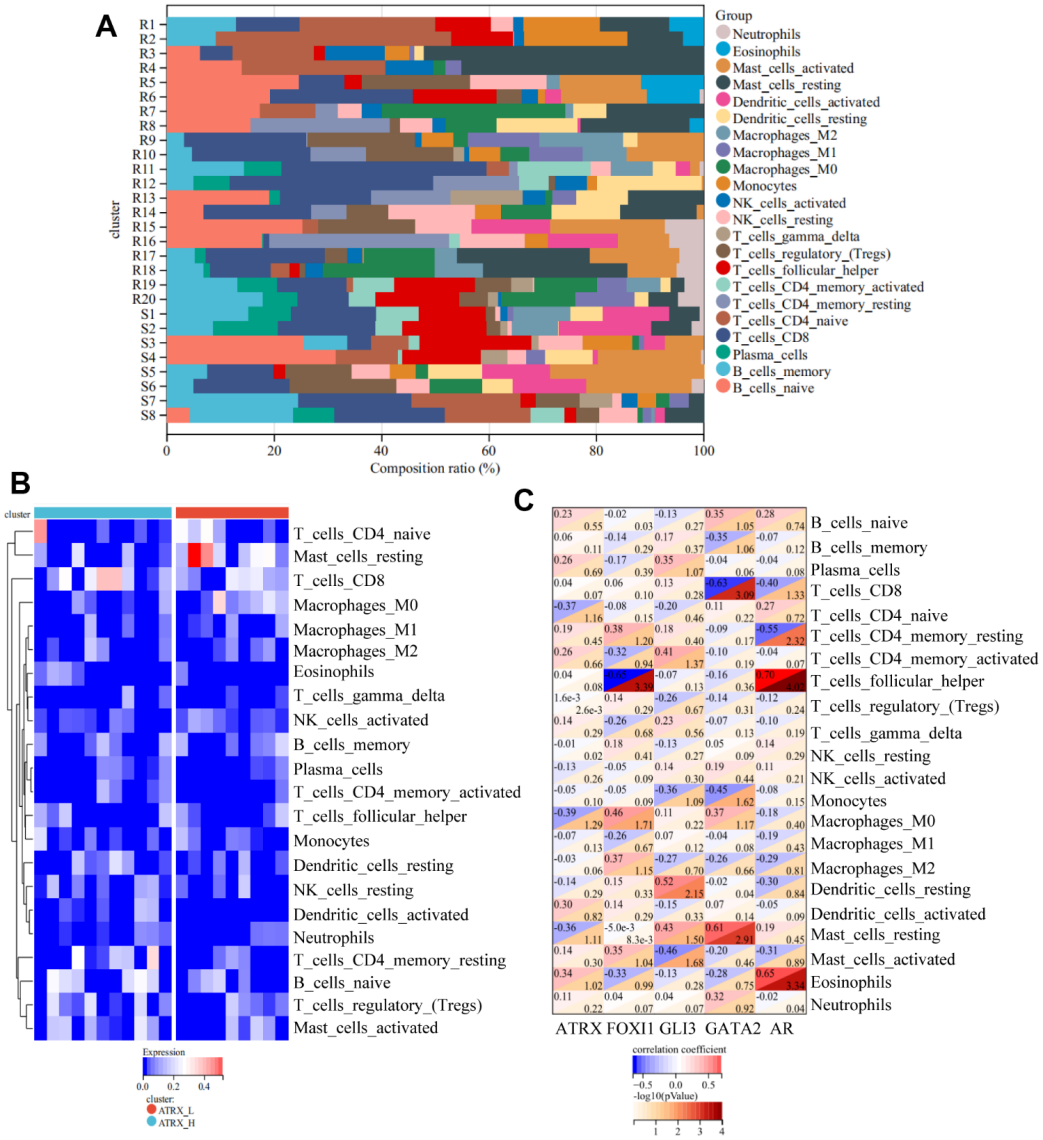
**Immune analyses for GSE22513.** (**A**) CIBERSORT analysis for 22 immunity cells. (**B**) ssGSEA revealed the difference in the 22 immunity cells between the ATRX-High and ATRX-Low groups. (**C**) Relationship between the five genes and immunity cells.

As the above findings suggested the importance of immune regulation in drug resistance, the immunity analysis for GSE22513, which contained tissue-related data and contained more information about immunity than the one from the cell level, was considered for analysis. CIBERSORT analysis revealed that the proportion of 22 immune cells in each case, wherein naïve B cells, naïve CD8 T cells, CD4 T cells and activated Dendritic cells were lost or reduced while resting CD4 memory T cells and resting Dendritic cells were increased in the resistance group (Figure 5A). Furthermore, resting CD4 memory T cells and resting Mast cells were strongly upregulated while activated Dendritic cells were significantly suppressed in resistance samples, which validated that immunity-regulation was inhibited in the resistance group (Supplementary Figure 1C). Moreover, ssGSEA revealed a significantly lower immune status in the ATRX-Low group than in the ATRX-High group (Figure 5B). Furthermore, ATRX showed a negative correlation with resting mast cells and M0 Macrophages, indicating that elevated ATRX levels could promote immunity. While AR, GLI3 and GATA2 were negatively correlated with follicular helper T cells, activated Mast cells, CD8 and CD4 memory resting T cells, suggesting that elevated TFs may inhibit immunity (Figure 5C). Therefore, these findings indicate that the role of ATRX was opposite to AR, GLI3 and GATA2 in immunity.

### Elucidating the potential mechanism of ATRX-mediated drug resistance in BC

**Figure 6:**
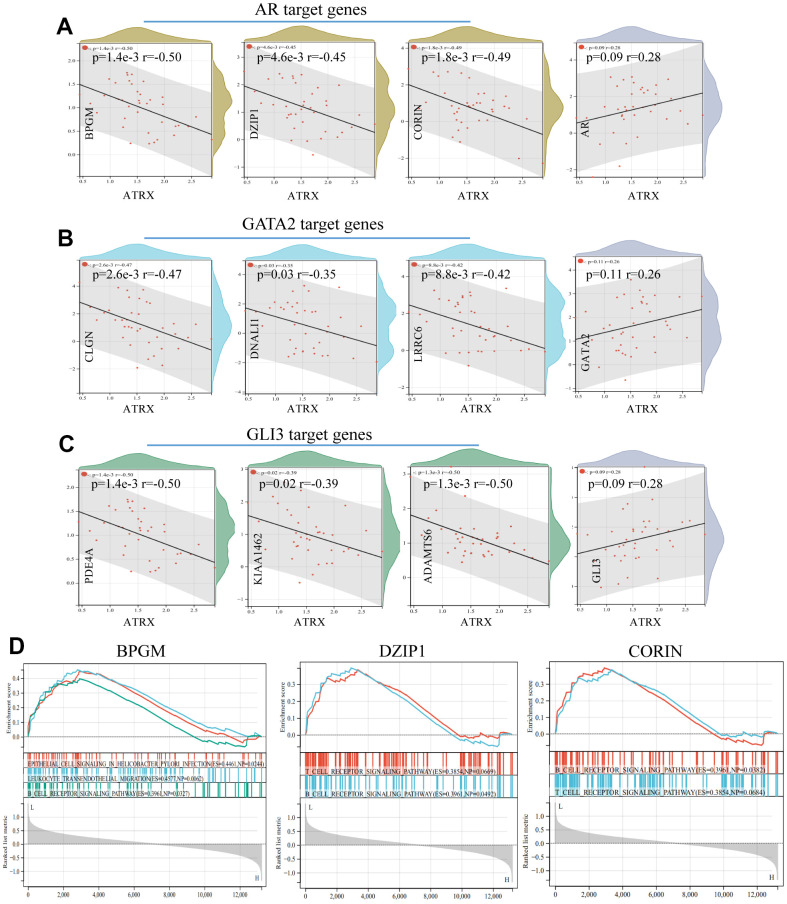
**Relationship between ATRX and target genes in GSE1.** The relationship between ATRX and the target genes of AR (**A**), GATA2 (**B**) and GLI3 (**C**). (**D**) GSEA analysis of AR’s target genes.

To further explore the potential mechanism of drug resistance in BC, we explored the relationship between ATRX and the three TFs in the network. We predicted and combined the target genes from JASPAR, ENCODE, CHEA and the MotifMap database. Notably, ATRX was significantly negatively correlated with certain DEGs, which were the three TFs’ target genes (BPGM, DZIP1 and CORIN as AR’s target genes; CLGN, DNALI1 and LRRC6 as GATA2’s target genes; PDE4A, KIAA1462 and ADAMTS6 as GLI3’s target genes). However, ATRX revealed no relationship with the three TFs at the RNA level (Figure 6A–6C). Moreover, GSEA illustrated that the low expression of the target genes promoted immunity regulation (Figure 6D).

**Figure 7:**
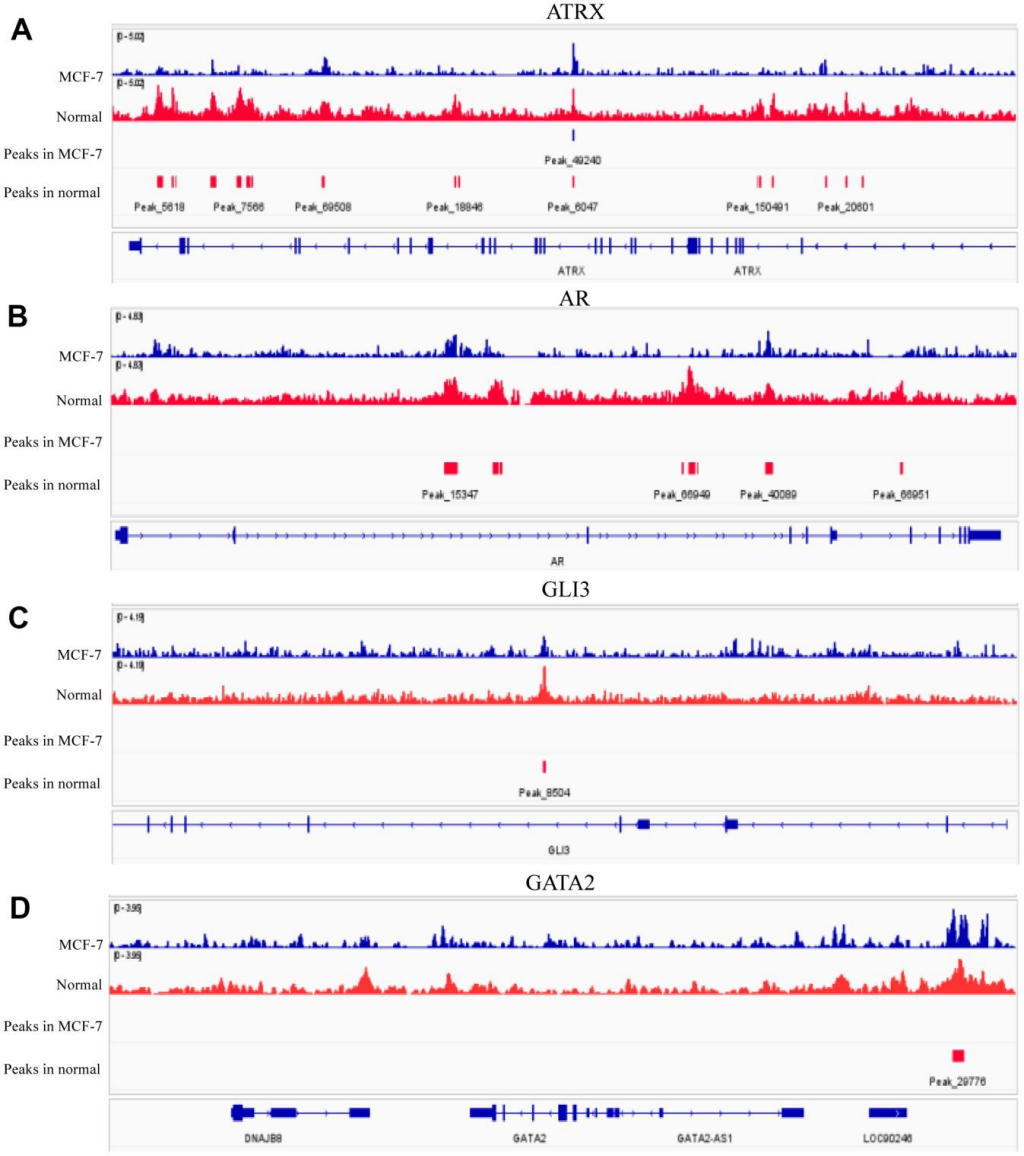
**H3K9me3 ChIP-seq for normal breast epithelium tissue and MCF-7 in ENCODE.** The signal intensities and peaks of H3K9me3 on ATRX (**A**), AR (**B**), GLI3 (**C**) and GATA2 (**D**) in normal breast epithelium tissues and 10 MCF-7 cells.

ATRX can bind to H3K9me3 to facilitate heterochromatin formation, which inhibits its transcription. Therefore, we compared H3K9me3 ChIP-seq data of normal breast epithelium tissues and MCF-7 from ENCODE Database, wherein both the signal intensities and the number of signal peaks on ATRX, AR, GLI3 and GATA2 were more abundant in normal tissues than that in MCF-7 (Figure 7). Furthermore, we determined the simple nucleotide variation data of BC samples from TCGA. Overall, missense mutations were the most common type. Moreover, a horizontal histogram revealed the frequent mutant genes in BC, namely TP53 (89.7%), ATRX (7.2%), BRCA2 (6.4%), BRCA1 (6.1%) and EGFR (3.4%) (Supplementary Figure 3A), indicating the downregulation of ATRX in BC drug resistance could be attributed to mutation. As ATRX can bind with AR directly, we predicted their potential interaction patterns, such as acetylation, ubiquitination, SUMOylation and so on (Supplementary Figure 3B). Moreover, decreased ATRX reduced the recruitment of H3K9me3 to AR, GATA2 and GLI3 to reduce the inhibition of transcription, resulting in the inhibition of immunity, promotion of the AR and PI3K-Akt signalling pathways, endocrine resistance and platinum drug resistance.

### Validating the expression of ATRX using the TMA of BC

**Figure 8:**
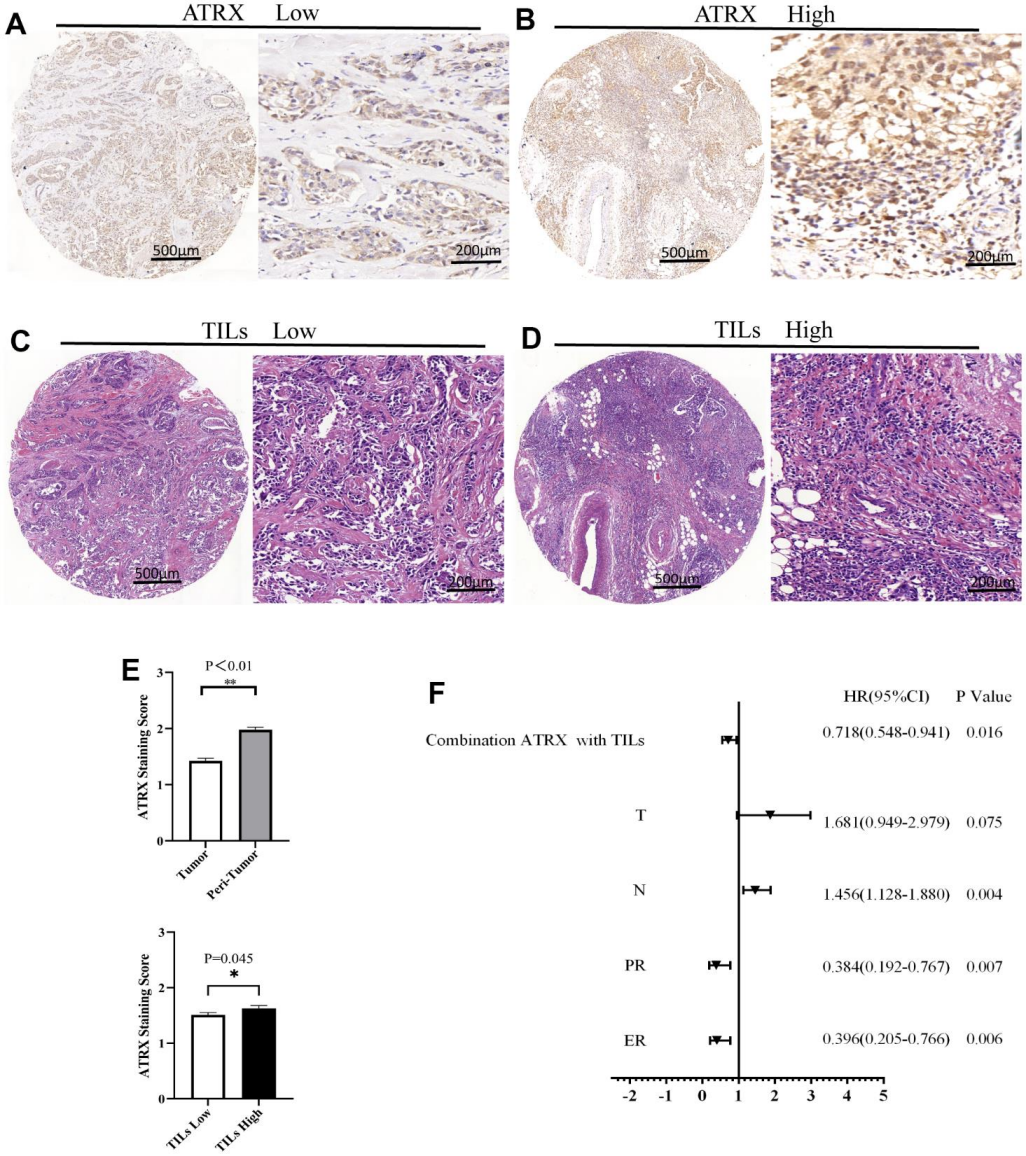
**ATRX and TILs on the TMA of BC.** Representative images for ATRX low (**A**) and ATRX high (**B**) in the samples. Representative images for TILs low (**C**) and TILs high (**D**) in the samples. The images of (**A**, **C**) came from the same sample, while the ones of (**B**, **D**) came from another sample. (**E**) Statistical analysis of ATRX expression. (**F**) Univariate analysis screened risk factors for OS in patients with BC.

IHC staining validated ATRX expression on the TMA. A representative TMA stained for ATRX is shown in Figure 8A, 8B. ATRX expression was localised in the nucleus of tumour cells but was abundant in the 136 tissues of the TMA, which were defined as ATRX-High group (Figure 8A and Table 1). However, ATRX staining was low in the other 108 tissues, which were defined as ATRX-Low group (Figure 8B and Table 1). Interestingly, certain tissues appeared the same tendency of ATRX and TILs (Figure 8C–8E). Next, we analysed the relationship between ATRX expression and clinicopathological factors, revealing a significant correlation between ATRX expression and the ER status (P = 0.008), PR status (P = 0.024), histologic grade (P = 0.035), TILs (P = 0.045), chemotherapy (P = 0.041), OS (P = 0.013) and DFS (P = 0.033) (Table 1).

**Table 1:** Characteristics of breast cancer patients and their ATRX expression level.

**Characteristics**	**All patients**		**ATRX Low**		**ATRX High**		**P-value**
	**n=244**		**n=108**		**n=136**		
	**No.**	**(%)**	**No.**	**(%)**	**No.**	**(%)**	
**Age at diagnosis (years)**							0.242
≤55	127	52.0	53	49.1	74	54.4	
>55	117	48.0	55	50.9	62	45.6	
**Menopausal status**							0.121
Pre-menopause	97	39.8	38	35.2	59	43.4	
Post-menopause	147	60.2	70	64.8	77	56.6	
**BMI(kg/m^2^)**							0.241
≤24	118	48.4	49	45.4	69	50.7	
>24	126	51.6	59	54.6	67	49.3	
**T**							0.916
T1	116	47.5	50	46.3	66	48.5	
T2	122	50.0	55	50.9	67	49.3	
T3	6	2.5	3	2.8	3	2.2	
**N**							0.528
N0	154	63.1	73	67.6	81	59.6	
N1	37	15.2	13	12.0	24	17.6	
N2	26	10.7	10	9.3	16	11.8	
N3	27	11.0	12	11.1	15	11.1	
**TNM**							0.923
I	83	34.0	38	35.2	45	33.1	
II	109	44.7	48	44.4	61	44.9	
III	52	21.3	22	20.4	30	22.0	
**ER**							**0.008**
Negative	120	49.2	63	58.3	57	41.9	
Positive	124	50.8	45	41.7	79	58.1	
**PR**							**0.024**
Negative	133	54.5	67	62.0	66	48.5	
Positive	111	45.5	41	38.0	70	51.5	
**HER2**							0.253
Negative	178	73.0	76	70.4	102	75.0	
Positive	66	27.0	32	29.6	34	25.0	
**Breast cancer subtype**							0.058
HR+/HER2+	29	11.9	11	10.2	18	13.2	
HR-/HER2+	37	15.2	21	19.4	16	11.8	
HR+/HER2-	96	39.3	34	31.5	62	45.6	
TNBC	82	33.6	42	38.9	40	29.4	
**Histologic grade**							**0.035**
1	15	6.1	2	1.9	13	9.6	
2	144	59.0	64	59.3	80	58.8	
3	85	38.9	42	38.9	43	31.6	
**TILs**							**0.045**
Low	147	60.2	72	66.7	75	55.1	
High	97	39.8	36	33.3	61	39.8	
**Lymphatic invasion**							0.174
Negative	149	61.1	70	64.8	79	58.1	
Positive	95	38.9	38	35.2	57	41.9	
**Vascular invasion**							0.137
Negative	219	89.8	100	92.6	119	87.5	
Positive	25	10.2	8	7.4	17	12.5	
**Nerve invasion**							0.456
Negative	238	97.5	106	98.1	132	97.1	
Positive	6	2.5	2	1.9	4	2.9	
**Chemotherapy**							0.249
<2	79	32.4	32	29.6	47	34.6	
≥2	165	67.6	76	70.4	89	65.4	
**Radiotherapy**							0.303
No	208	85.2	94	87.0	114	83.8	
Yes	36	14.8	14	13.0	22	16.2	
**Targeted therapy**							0.150
No	219	89.8	94	87.0	125	91.9	
Yes	25	10.2	14	13.0	11	8.1	
**Endocrinotherapy**							0.141
No	164	67.2	77	71.3	87	64.0	
Yes	80	32.8	31	28.7	49	36.0	
**OS**							**0.013**
Survival	211	86.5	87	80.6	124	91.2	
Death	33	13.5	21	19.4	12	8.8	
**DFS**							**0.033**
No	203	83.2	84	77.8	119	87.5	
Yes	41	16.8	24	22.2	17	12.5	

**Figure 9:**
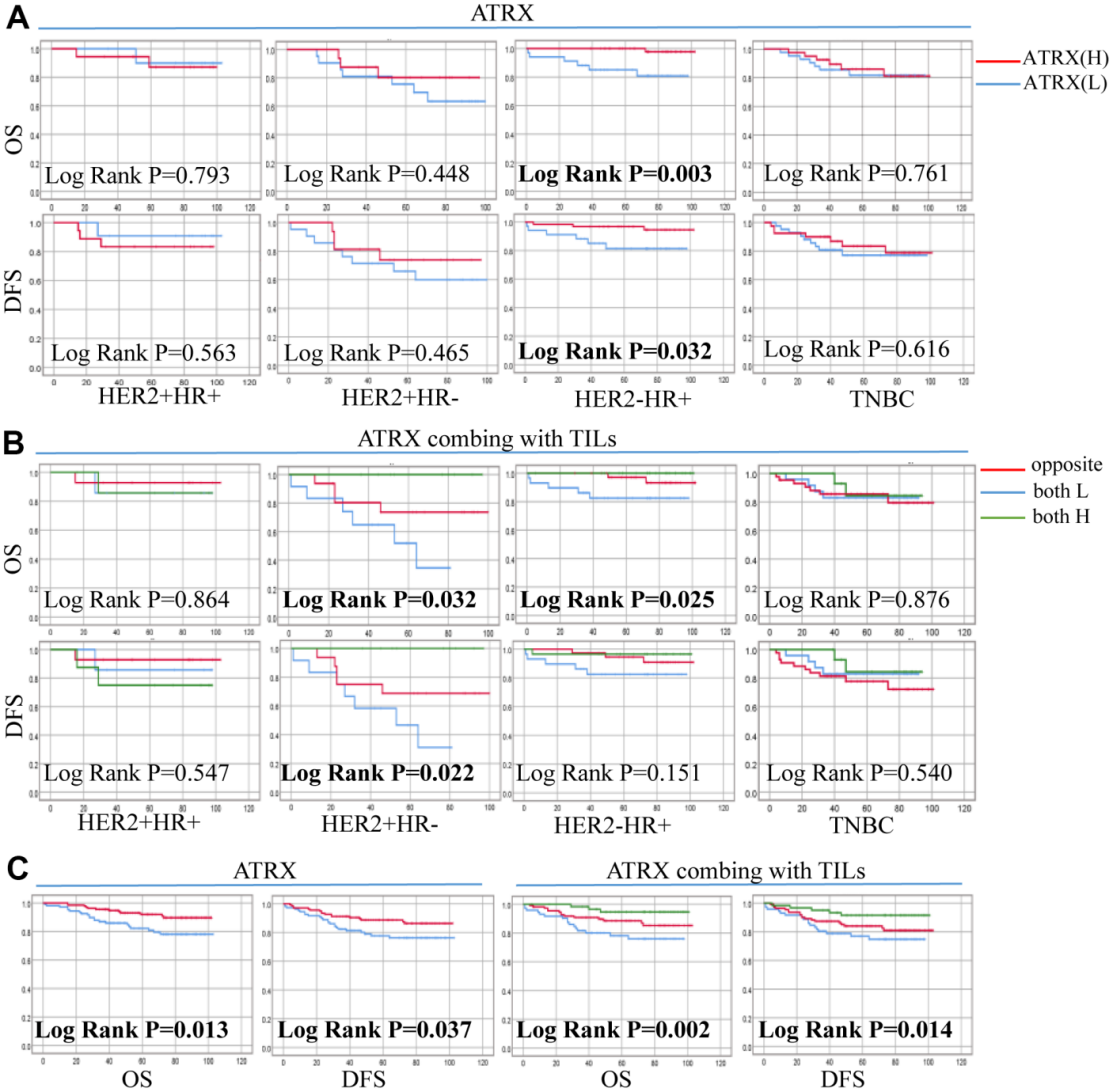
**Correlation between ATRX expression, TILs level and survival outcomes in patients with on the TMA.** (**A**) Correlation between ATRX and OS, DFS in the four BC subtypes. (**B**) Correlation between ATRX combined with TILs and OS, DFS in the four BC subtypes. (**C**) Correlation between ATRX (or ATRX combining with TILs) and OS, DFS in patients with BC. ATRX(H): ATRX-High expression group, ATRX(L): ATRX-Low expression group, Opposite: the group with ATRX expression tendency opposited to TILs, both H: both ATRX- and TILs-High group, both L: both ATRX- and TILs-Low group.

The mean follow-up time for OS and DFS were 66.93 months (range, 1–103 months) and 64.07 months (range, 1-103 months), respectively. During the follow-up period, 16.8% (41 of 244) of the patients had recurrence and/or metastasis whereas 13.5% (33 of 244) died. Univariate and Multivariate analyses of ATRX associated with OS in patients revealed that ER, T, N and combination ATRX with TILs significantly correlated with OS (Table 2). Similarly, PR, T and combination ATRX with TILs correlated significantly with DFS (Table 3). Survival analyses were performed using the Kaplan-Meier method with a log-rank test. The results showed that ATRX expression was positive with OS (P = 0.013) and DFS (P = 0.037) in patients with BC while the combination of ATRX with TILs showed a better correlation with both OS (P = 0.002) and DFS (P = 0.014). Furthermore, ATRX was significantly correlated with OS (P = 0.003) and DFS (P = 0.032) of patients with HER2-/HR+ BC. Additionally, the combination of ATRX with TILs showed a correlation with OS (P = 0.025) in patients with HER2-/HR+ BC (Figure 9).

**Table 2:** Univariate and multivariate analysis of ATRX associated with OS in patients with BC.

**Variables**	**Univariate analysis**			**Multivariate analysis**		
	**HR**	**95%CI for HR**	***P***	**HR**	**95%CI for HR**	***P***
**ER**	0.361	0.172-0.760	**0.007**			**0.025**
Negative				2.418	1.119-5.225	
Positive				Ref.		
**PR**	0.386	0.179-0.832	**0.015**			
Negative						
Positive						
**Histologic grade**	1.642	0.888-3.035	0.114			
1						
2						
3						
**T**	1.906	1.017-3.574	**0.044**			**0.031**
T1				0.165	0.042-0.643	0.009
T2				0.204	0.056-0.748	0.016
T3				Ref		
**N**	1.279	0.955-1.711	0.098			**0.047**
N0				0.428	0.187-0.982	0.045
N1				0.374	0.111-1.259	0.112
N2				0.081	0.010-0.673	0.020
N3						
**Combination ATRX with TILs**	0.469	0.283-0.776	**0.003**			**0.016**
ATRX&TILs (both L)				5.660	1.609-19.911	0.007
ATRX&TILs (opposite)				2.925	0.831-10.304	0.095
ATRX&TILs (both H)				Ref		

**Table 3:** Univariate and multivariate analysis of ATRX associated with DFS in patients with BC.

**Variables**	**Univariate analysis**			**Multivariate analysis**		
	**HR**	**95%CI for HR**	***P***	**HR**	**95%CI for HR**	***P***
**ER**	0.396	0.205-0.766	**0.006**			
Negative						
Positive						
**PR**	0.384	0.192-0.767	**0.007**			**0.027**
Negative				2.228	1.096-4.526	
Positive				Ref.		
**Histologic grade**	1.739	1.000-3.022	0.050			
1						
2						
3						
**T**	1.681	0.949-2.979	0.075			**0.026**
T1				0.159	0.052-0.487	0.001
T2				0.171	0.057-0.514	0.002
T3				Ref.		
**N**	1.327	1.025-1.719	**0.032**			
N0						
N1						
N2						
N3						
**Combination ATRX with TILs**	0.587	0.380-0.906	**0.016**			**0.041**
ATRX&TILs (both L)				3.292	1.203-9.005	0.020
ATRX&TILs (opposite)				2.094	0.773-5.669	0.146
ATRX&TILs (both H)				Ref		

**Figure 10:**
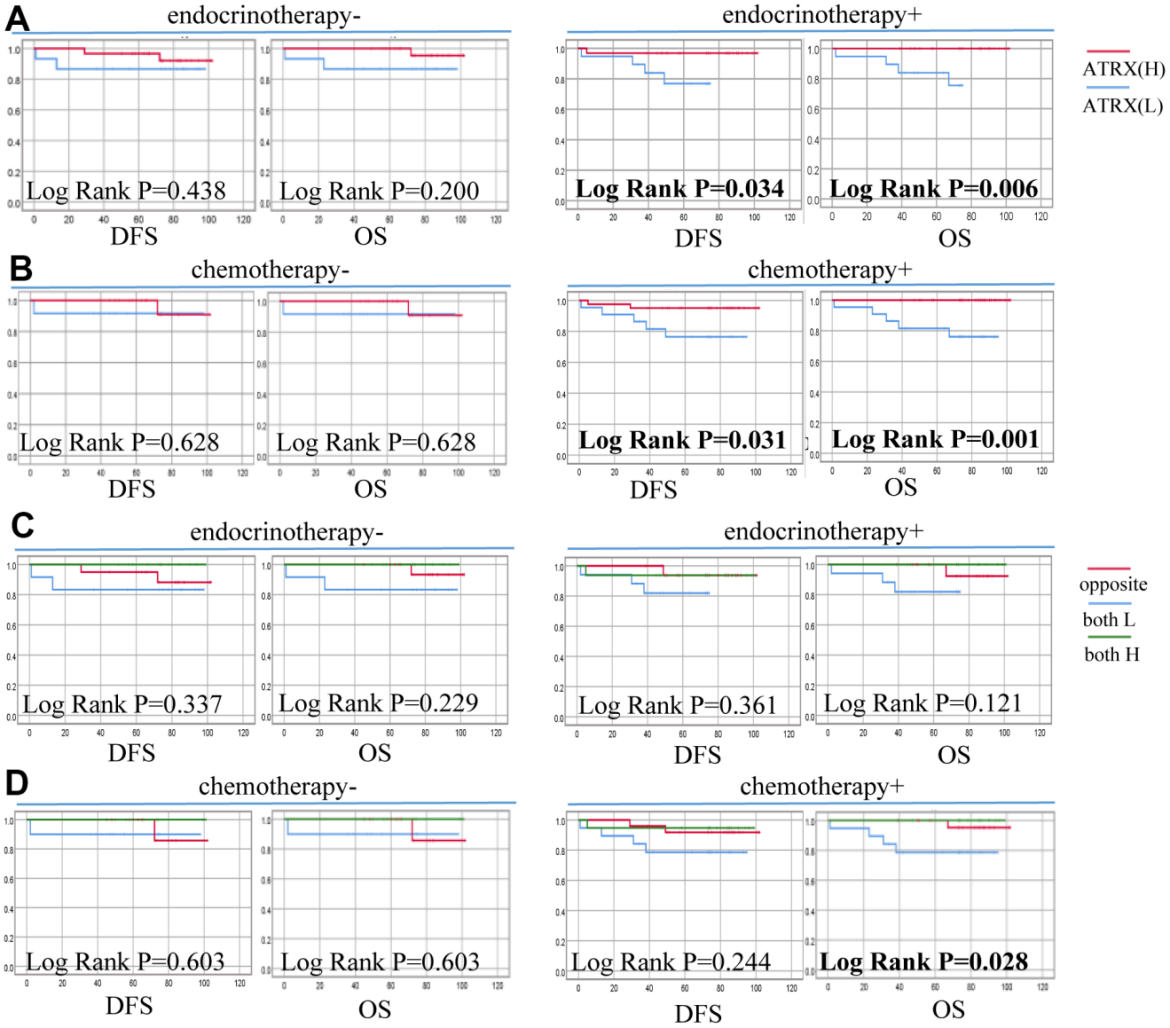
**Correlation between ATRX expression, TILs level and survival outcomes of patients with HER2-/HR+ BC on the TMA.** (**A**) Correlation between ATRX and OS, DFS in patients with HER2-/HR+ BC who received endocrinotherapy or did not receive endocrinotherapy. (**B**) Correlation between ATRX and OS, DFS in patients with HER2-/HR+ BC who received chemotherapy or did not receive chemotherapy. (**C**) Correlation between ATRX combined with TILs and OS, DFS in patients with HER2-/HR+ BC who received endocrinotherapy or did not receive endocrinotherapy. (**D**) Correlation between ATRX combined with TILs and OS, DFS in patients with HER2-/HR+ BC who received chemotherapy or did not receive chemotherapy. ATRX(H): ATRX-High expression group, ATRX(L): ATRX-Low expression group, Opposite: the group with ATRX expression tendency opposited to TILs, both H: both ATRX- and TILs-High group, both L: both ATRX- and TILs-Low group.

In particular, the low expression of ATRX predicted poor DFS of the HER2-/HR+ BC subgroup who underwent endocrine (P = 0.034) or chemotherapy (P = 0.031) treatment. Similarly, suppressed ATRX revealed a bad OS for the same subgroup who received endocrine (P = 0.006) or chemotherapy (P = 0.001) treatment (Figure 10). Univariate analysis was performed for screening of risk factors, wherein combination ATRX with TILs (HR = 0.718, P = 0.016), PR (HR = 0.384, P = 0.007), ER (HR = 0.396, P = 0.006) were negatively correlated with OS while N (HR = 1.456, P = 0.004) was positively correlated with OS (Figure 8F).

### Constructing a nomogram to predict survival in patients with BC

**Figure 11:**
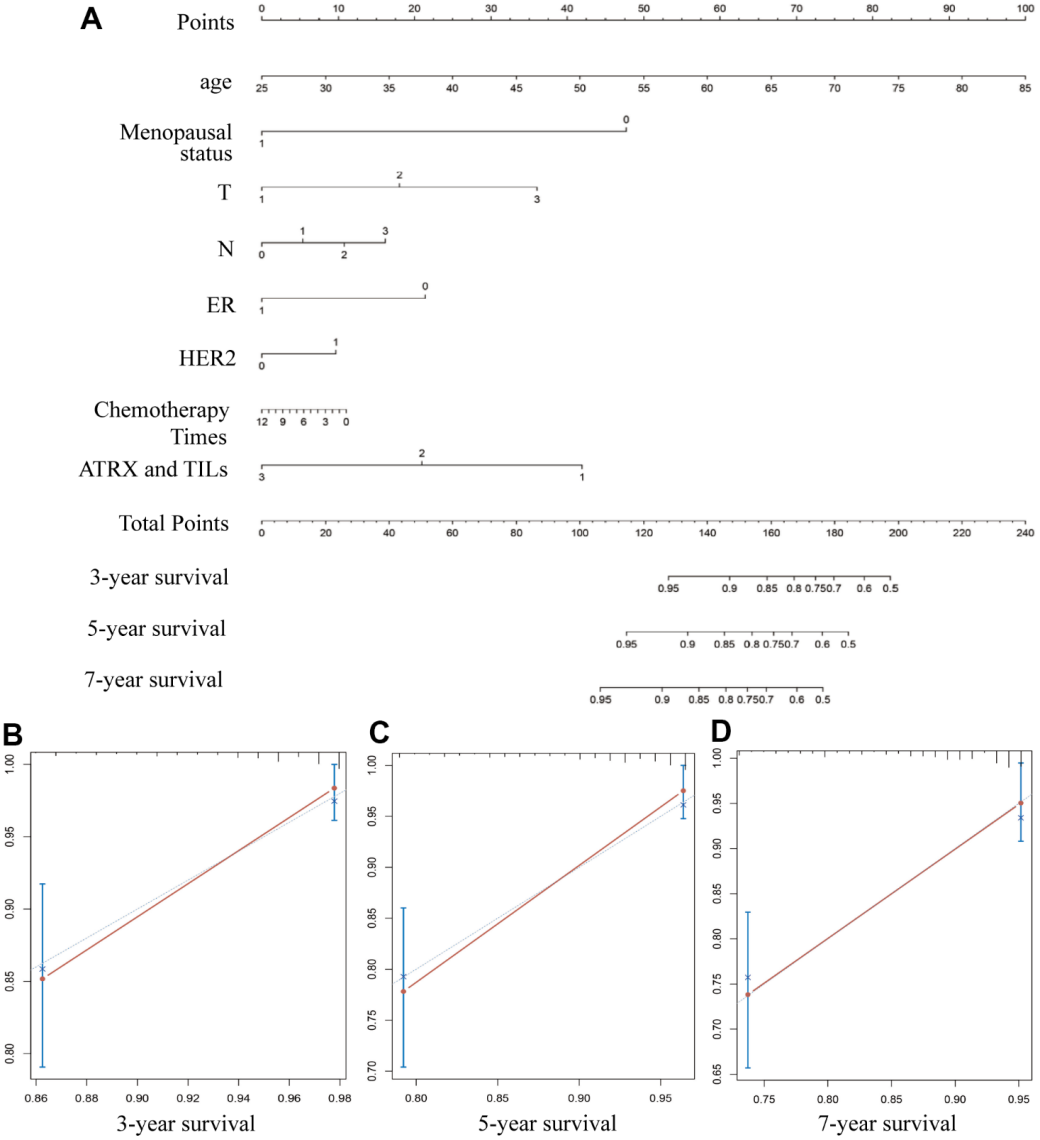
**Constructing a nomogram for prognosis prediction.** (**A**) The predicted 3-, 5- and 7-year survival rates of patients with BC are based on the prognostic nomogram. (**B**–**D**) C-index showed the concordances between predicted and observed 3-, 5- and 7-year survival rates based on the nomogram after bias corrections.

Nomogram survival prediction plots are commonly used to predict patient survival, with scores reflecting the values of several prognostic variables. Thus, we constructed a nomogram to estimate the probability of survival at 3, 5 and 7 years. The C-index value of the nomogram was 0.79. The calibration curves depicting the actual and nomogram-predicted survival at 3, 5 and 7 years were within the reference limits, reminding the nomogram based on our prognostic signature is precise and reliable (Figure 11). Moreover, receiver operating characteristics (ROC) analysis evaluated the predictive ability of ARTX in the TMA data. The AUC of ATRX for predicting the 3-year OS (AUC =0.842, P = 0.042) and the 5-year OS (AUC =0.848, P = 0.021) of the HER2-/HR+ BC patients who underwent endocrinotherapy were ideal (Supplementary Figure 4A). The similar good performance of ATRX was also found for the HER2-/HR+ BC patients who underwent chemotherapy (Supplementary Figure 4B).

### Drug-gene interactions

**Figure 12:**
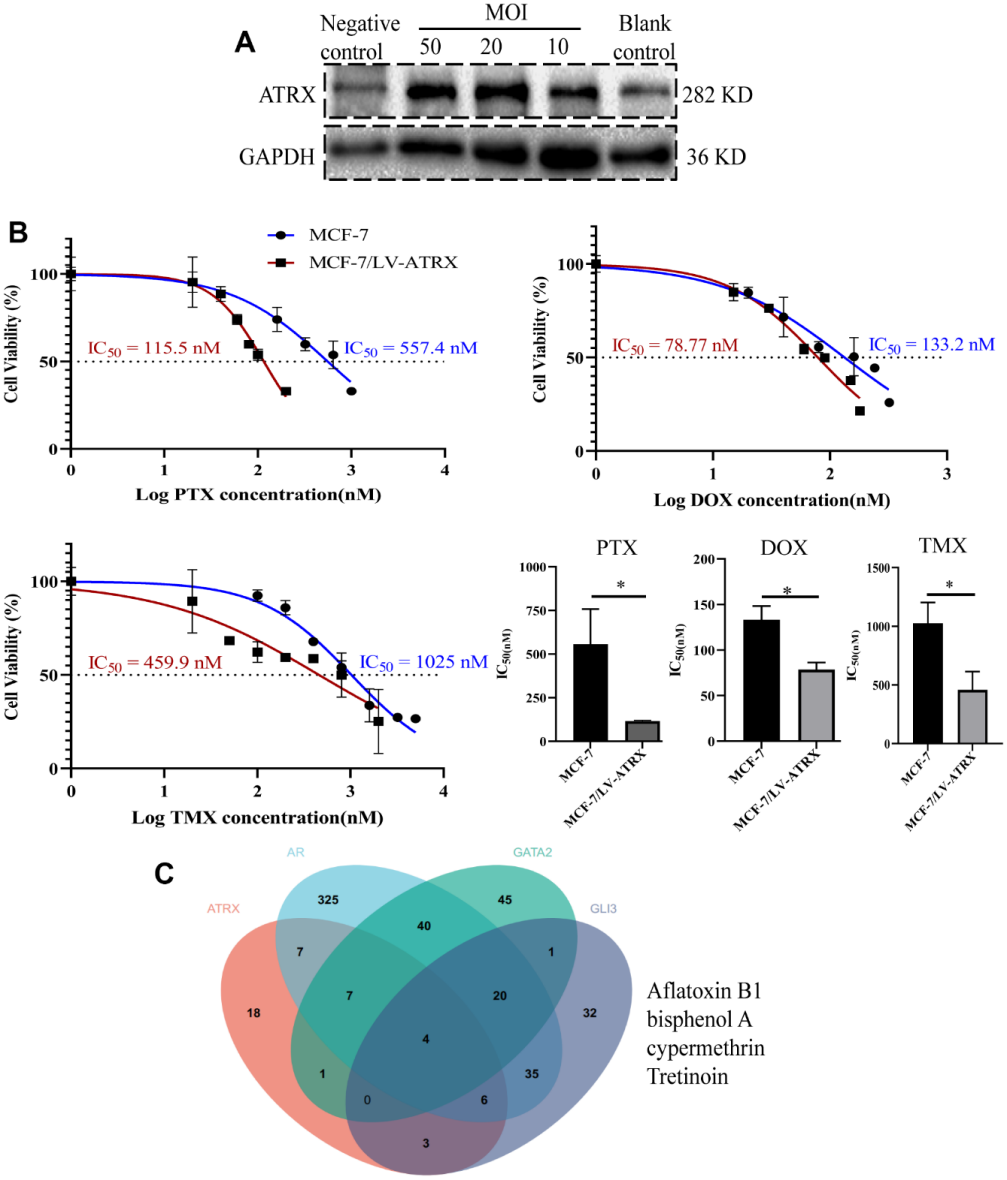
**Drug sensitivity analysis and drug prediction.** (**A**) Western blot showed the expression of ATRX when lentivirus was transfected in different concentrations (MOI=10, 20, 50) in MCF-7 cell. Lentivirus without ATRX was served as negative control, and that without lentivirus vector was served as blank control. (**B**) IC_50_ of PTX, DOX and TMX (for 48 h) was detected on MCF-7 cell and MCF-7 cell with overexpression of ATRX. The experiment was repeated three times. (**C**) Venn diagram detected the predicted drugs in CTDbase, which can promote ATRX and also inhibit AR, GLI3 and GATA2.

In order to verify the role of ATRX in the first-line drugs sensitivity to HER2-/HR+ BC, the half maximal inhibitory concentration (IC_50_) of PTX, DOX and TMX on MCF-7 cell and MCF-7 cell with lentivirus-mediated ATRX overexpression were detected. Westernblot showed when the multiplicity of infection (MOI) of lentivirus was 50 in MCF-7 cell, the expression of ATRX was increased significantly (Figure 12A). Therefore, we chose this MOI value for the following experiment. Interestingly, we found overexpression of ATRX significantly inhibited the IC_50_ of the three first-line drugs on MCF-7 cell, especially the IC_50_ of PTX and TMX dropped sharply below 50% (Figure 12B).

As ATRX, AR, GATA2 and GLI3 are vital to gene mediated drug resistance, the CTDbase database was used to obtain potential drugs. A total of four drugs were identified to promote ATRX and inhibit AR, GLI3 and GATA2. Among the identified drugs, bisphenol and tretinoin were considered ideal drugs (Figure 12C).

## DISCUSSION

As BC is a heterogeneous disease, multifactorial resistance mechanisms are involved in the different subtypes [22]. Various mechanisms for HER2-/HR+ BC, such as the upregulation of membrane receptor kinases or dysregulation of ER have been reported. However, the alteration of individual molecules could not address this challenge, suggesting that drug resistance could be attributed to the dysregulation of molecular networks. Notably, epigenetic regulation, involving many genes, is an important mechanism for drug resistance [23].

Based on WGCNA and the PPI interaction network, we identified ATRX as a hub gene. ATRX is mainly involved in the scope of nuclear functions [24–27]. Importantly, ATRX combines with DAXX, forming heterochromatin characterized by H3K9me3 and reducing the accessibility of chromatin to TFs and chromatin remodels, resulting in transcriptional repression [28]. Consistent with previous reports, we found that ATRX was downregulated significantly in the drug resistant group. Moreover, the H3K9me3-ChIP signal of ATRX in BC tissues was much lower than that in normal tissues. Mutation of ATRX was a common phenomenon in numerous tumours and contributed to tumour development [29]. Additionally, the frequency of ATRX mutation was higher than BRCA in BC samples. These results collectively suggested that the function of maintaining genome stability was damaged owing to the mutation of ATRX in BC.

ATRX recruits H3K9me3 to compact cis-regulatory elements bound by TFs, thereby abolishing the downstream gene programs. Based on the intersection of ATRX associated genes, TFs and DEGs, we found that ATRX, AR, GLI3 and GATA2 can regulate each other as a transcriptional network in drug resistance in BC. Notably, AR not only interacts with ATRX directly but also interacts with the highest number of genes in this network. Increasing pieces of evidence have demonstrated the importance of AR in BC, which is expressed in up to 90% of ER+ BC [30]. AR overexpression was demonstrated to be a key marker of aromatase inhibitors resistance and Tamoxifen-resistance [20, 31–33]. Similarly, this study also verified that AR expression was significantly increased in drug resistance. GATA2 is a critical pioneer TF for AR, trans-activated IGF2 to mediate taxane resistance in prostate cancer or promote chemoresistance in gastric cancer via the EGFR signalling pathway [20, 34]. Moreover, GLI3 interacts with AR to enrich AR-dependent gene expression, leading to the castration-resistant growth of prostate tumours [35]. Additionally, GATA2 and GLI3 were upregulated in the resistance group. GO and KEGG analysis for these genes in the network showed that the AR signalling pathway, negative regulation of transcription by RNA polymerase II and basal transcription factors were enriched. Notably, the immunity-regulation signalling pathway was also enriched, further validating these findings.

Immunosuppression is an important factor in the development of drug resistance [36]. For example, the depletion of T lymphocytes [37] and dysfunction of NK cells [38] have been reported to play a role in the drug resistance of tumours [39]. We found that immunity-regulation was inhibited in the resistance group whereas the ATRX-Low group showed a significantly low immune status. Conversely, elevated AR, GLI3 and GATA2 suppressed immunity. We considered the potential reasons for the phenomenon: ATRX recruited H3K9me3 to AR, GLI3 and GATA2 and consequently inhibited the transcription of the target genes. Accordingly, the H3K9me3-ChIP signal peaked on AR, GLI3 and GATA2 when ATRX was upregulated in normal tissue. Additionally, the peaks decreased when ATRX was downregulated in BC. Furthermore, the expression tendency of ATRX was converse to the DEGs.

ATRX suppression was associated with immunity inhibition and multiple drug resistance signalling pathways from the database analysis, which were further validated using the TMA of BC. Interestingly, ATRX was significantly correlated with TILs, chemotherapy and HR status. As TILs have been proven to evaluate chemotherapy in BC by reflecting immune response [40], we combined ATRX with TILs and found their combination allowed for the better prediction of OS and DFS in BC. Furthermore, ATRX displayed a significant correlation with OS and DFS in the BC HER2-/HR+ subtype. Particularly, the low expression of ATRX predicted poor OS and DFS in the patients with the HER2-/HR+ BC subtype who underwent endocrine or chemotherapy treatment. Thus, we speculated that inhibited ATRX mediated the endocrine or chemotherapy resistance for the HER2-/HR+ subgroup through the loss of regulating the TF network.

This study has limitations. Our study is retrospective and determines ATRX expression alone. Hence, more experiments should be employed to elucidate the mechanism of drug resistance. In brief, we identified ATRX as an efficient predictive biomarker for endocrinotherapy and chemotherapy resistance in HER2-/HR+ BC. It acts by suppressing the AR, GLI3 and GATA2 TF networks. This research is the first, to the best of our knowledge, to report the importance of ATRX in HER2-/HR+ BC, providing new insight for clinicians to combat drug resistance.

## MATERIALS AND METHODS

### GEO data

As doxorubicin, paclitaxel and antiestrogen therapy are first-line anti-tumour drugs in BC, three microarray expression profiles on drug resistance in BC (GSE24460, GSE22513 and GSE8562) were downloaded from the Gene Expression Omnibus (GEO) database. The details are presented in Supplementary Table 1. The GSE24460 dataset (https://www.ncbi.nlm.nih.gov/geo/query/acc.cgi?acc=GSE24460) consists of two MCF-7 doxorubicin Resistance cells vs two MCF-7 cells (Sensitive). In GSE22513 (https://www.ncbi.nlm.nih.gov/geo/query/acc.cgi?acc=GSE22513), 20 non-pCR breast biopsies (paclitaxel) vs eight pCR breast biopsies (paclitaxel) samples were present, and we defined non-pCR breast biopsy as the resistant group and pCR breast biopsy as the sensitive group. The GSE8562 (https://www.ncbi.nlm.nih.gov/geo/query/acc.cgi?acc=GSE8562) dataset consisted of three MCF-7/X-box binding protein 1 (XBP1) (Estrogen Resistance) cells vs three MCF-7 cells (Sensitive), where XBP1 is a well-known endocrine resistant gene in BC [41, 42]. Due to the limited sample size, we merged the three datasets into a singular large dataset (named GSE1) and corrected the batch effect using the SangerBox 3.0 platform.

### Bioinformatics analysis

Limma analysis screened DEGs of the GEO data. The absolute value was identified |FC| > 1.2 and Benjamini–Hochberg-adjusted P < 0.05. GO and KEGG analyses were performed using the cluster profiler package in R in the SangerBox 3.0 platform. P < 0.05 was set as the cut-off criterion. Weighted Correlation Network Analysis (WGCNA) was performed in the SangerBox 3.0 platform. Additionally, interactive relationships and PPI networks of the DEGs were evaluated using the Network Analyst Database and STRING database. Human transcription factors (TFs) were downloaded from the human transcription factors database (http://humantfs.ccbr.utoronto.ca/allTFs.php). CIBERSORT algorithm analysed the proportion of 22 immune cells in resistant and sensitive samples, and the difference was compared using a t-test in the SangerBox 3.0 platform. Pearson’s correlation analysis calculated the correlation coefficient between immune cell infiltration and TFs. ssGSEA detected the different distribution of the 22 immunity cells between the ATRX-Low and ATRX-High groups using the SangerBox 3.0 platform. Furthermore, GSEA was used to associate genes with possible pathways in the SangerBox 3.0 platform. A false discovery rate < 0.5 and P < 0.05 were used as the criteria for statistical significance. Additionally, the trimethylation of histone H3 at lysine 9 (H3K9me3) ChIP-seq of normal breast epithelium tissue (female adult breast epithelium tissue) (https://www.encodeproject.org/experiments/ENCSR936LAH/) and MCF-7 (https://www.encodeproject.org/experiments/ENCSR999WHE/) in ENCODE were analysed, wherein ENCODE3 GRCh38 processed data were downloaded and normalised to compare the signal intensities and visualised using IGV.2.14.1 software. The mutation profiling in BC samples obtained from TCGA was presented in the SangerBox 3.0 platform. The potential interaction mechanism of AR and ATRX was predicted using the cBioPortal database. Drug-gene interactions were performed on the Network Analyst Database.

### Patient information and tissue microarray

Human experiments were performed according to the ethical standards of the Helsinki Declaration and the China Ministry of Health’s ‘Ethical Review of Human Biomedical Research (Tentative, 2007)’. Written informed consent was obtained from all participants. Primary BC samples were obtained from the Hospital Affiliated with Nantong University between 2012 and 2017. We collected formalin-fixed paraffin-embedded surgery tissues from 244 patients with BC along with their complete clinicopathological data for preparation of tissue microarray (TMA). The diagnosis had given by two pathologists who were blinded to the clinicopathological data. Samples were classified into the following BC subtypes: HER2+/HR+, HER2+/HR-, HER2-/HR+ and TNBC.

### Immunohistochemical (IHC) analysis

TMA was incubated with a primary antibody against a 1:200 dilution of ATRX (Rabbit-anti-human, Abcam) overnight at 4° C after the dewaxing and blocking of endogenous peroxides of the tissues. Visualisation of the antibody complex was achieved through a diaminobenzidine reaction, resulting in the brown staining of the cell nucleus. TMA was counterstained by Meyer’s haematoxylin. IHC staining was scored by pathologists based on the intensity of staining in the tumour cell nucleus from three hot spots of each tissue. The intensity of staining was scored as 0, negative; 1, weak; and 3, strong. We defined IHC staining score 0 and 1 as low ATRX expression and score 3 as high ATRX expression.

### Assessment of tumour-infiltrating lymphocytes (TILs)

Hematoxylin eosin staining was used to stain the TMA. At x200 magnification, the percentage of the area containing TILs in the tumour nest and stroma to the total tumour area was calculated, and its mean value was defined as the TILs ratio. The proportions of TILs ≤ 5%, between 6% and 49% and ≥50% were considered as negative, weak and high TILs proportion groups, respectively. Moreover, we defined the negative and weak TILs groups as low TILs while the high TILs group stayed the same.

### Western blot

The radio immunoprecipitation assay (Beyotime Biotechnology, Shanghai, China) lysis buffer supplemented with PMSF and protease inhibitor was used to extract total cellular protein. Protein was separated by 7.5% SDS-PAGE (Beyotime Biotechnology, China), transferred to polyvinylidene fluoride membrane, and sealed with 5% skim milk at room temperature. The membrane was sealed with diluted ATRX specific antibody (ab97508, Abcam, UK) at 4° C overnight, washed and incubated with secondary antibody at room temperature. After cleaning the membrane, enhanced chemiluminescence (Tanon, Shanghai, China) was used to detect protein bands.

### Cell viability assay

To determine the IC_50_, cell viability was assessed by CCK-8 (Vazyme, Nanjing, China) analysis. Cell suspensions of MCF-7 and MCF-7 with lentivirus-mediated ATRX overexpression, at a concentration of 5×10^4^ cell/100 μL, were seeded in a 96-well plate. The next day, cells were treated with different concentrations of DOX, PTX or TMX for 48 hours. Absorbances were assessed by detecting OD450 with an automatic spectrophotometer.

### Statistical analysis

SPSS V17 software was used to analyse GEO data or experimental data. IC_50_ was analysed by Graphpad Prism. Correlations between ATRX expression and clinicopathological characteristics were analysed using the χ^2^ test. We defined OS as the time from the date of the primary surgery until the date of death or the last follow-up. DFS was defined as the time from the date of the primary surgery to the date of recurrence, which indicated locoregional recurrence, distant metastasis or death from any cause. Kaplan-Meier detected survival analysis, and the log-rank test estimated associations between variables and survival. Univariable and Multivariable Cox regression models identified significant prognostic factors. Validation of the nomogram was performed based on the primary group. The concordance index (C-index) assessed the discrimination of the nomogram. A calibration curve detected the calibration. Notably, the curve was corroborated with 1000 resamples conducted for validation. P < 0.05 was considered significant.

## Supplementary Material

Supplementary Figures

Supplementary Tables
