# Supplementary material for: ATRX is a predictive marker for endocrinotherapy and chemotherapy resistance in HER2-/HR+ breast cancer through the regulation of the AR, GLI3 and GATA2 transcriptional network
Source: Aging (Albany NY). 2023 Dec 20;15(24):14996–5024. doi: 10.18632/aging.205327 (PMC10781474; doi:10.18632/aging.205327)
Supplement: Supplementary Figures [file aging-15-205327-s001.pdf]

SUPPLEMENTARY FIGURES

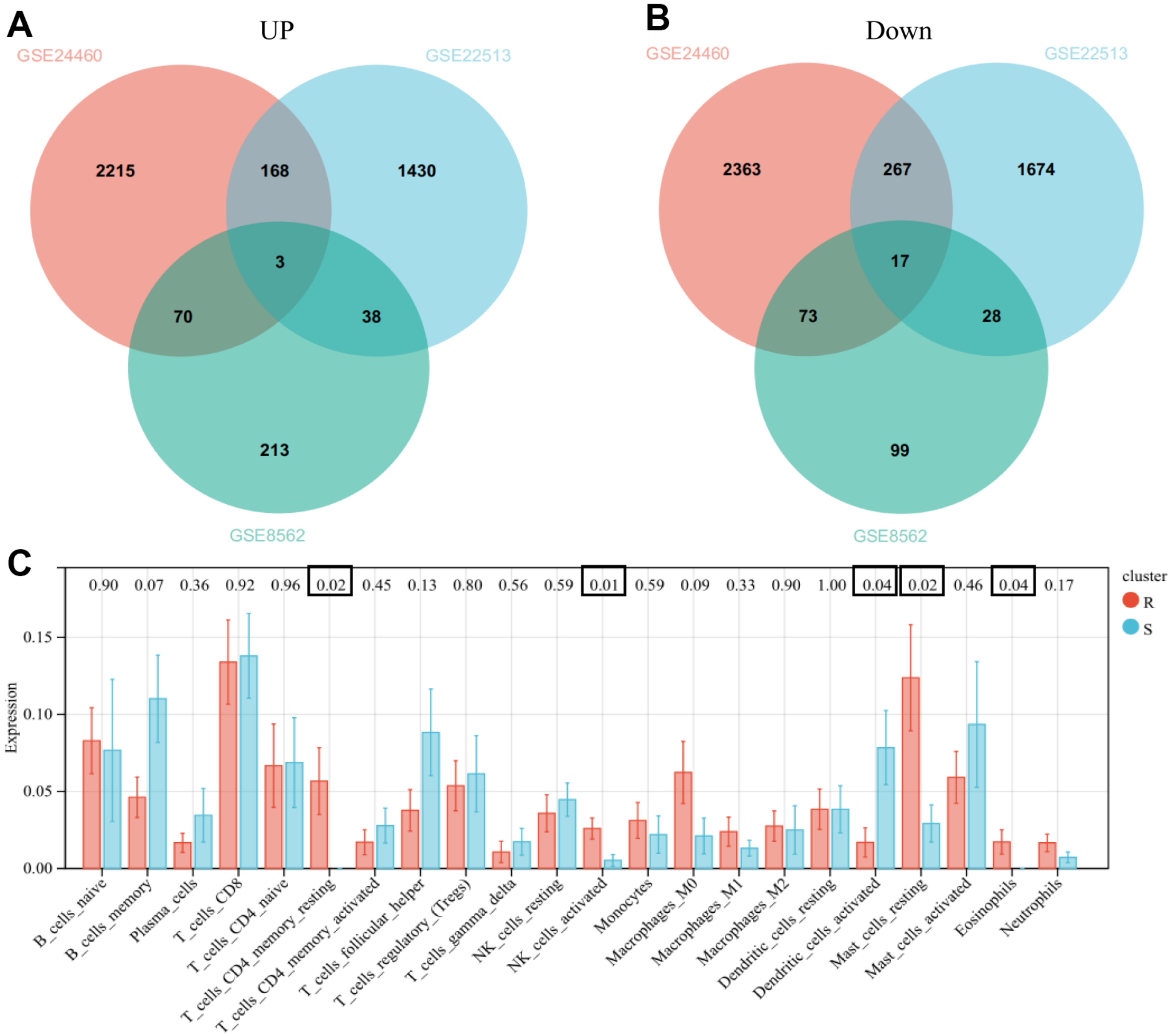

Supplementary Figure 1. Common DEGs from GSE24460, GSE862, GSE22513. Venn diagram detected the up-regulated (**A**) or down-regulated (**B**) common DEGs from the 3 GEO data. (**C**) Different distribution of immune cell in resistant and sensitive tissues in GSE22513. black frame:P<0.05.

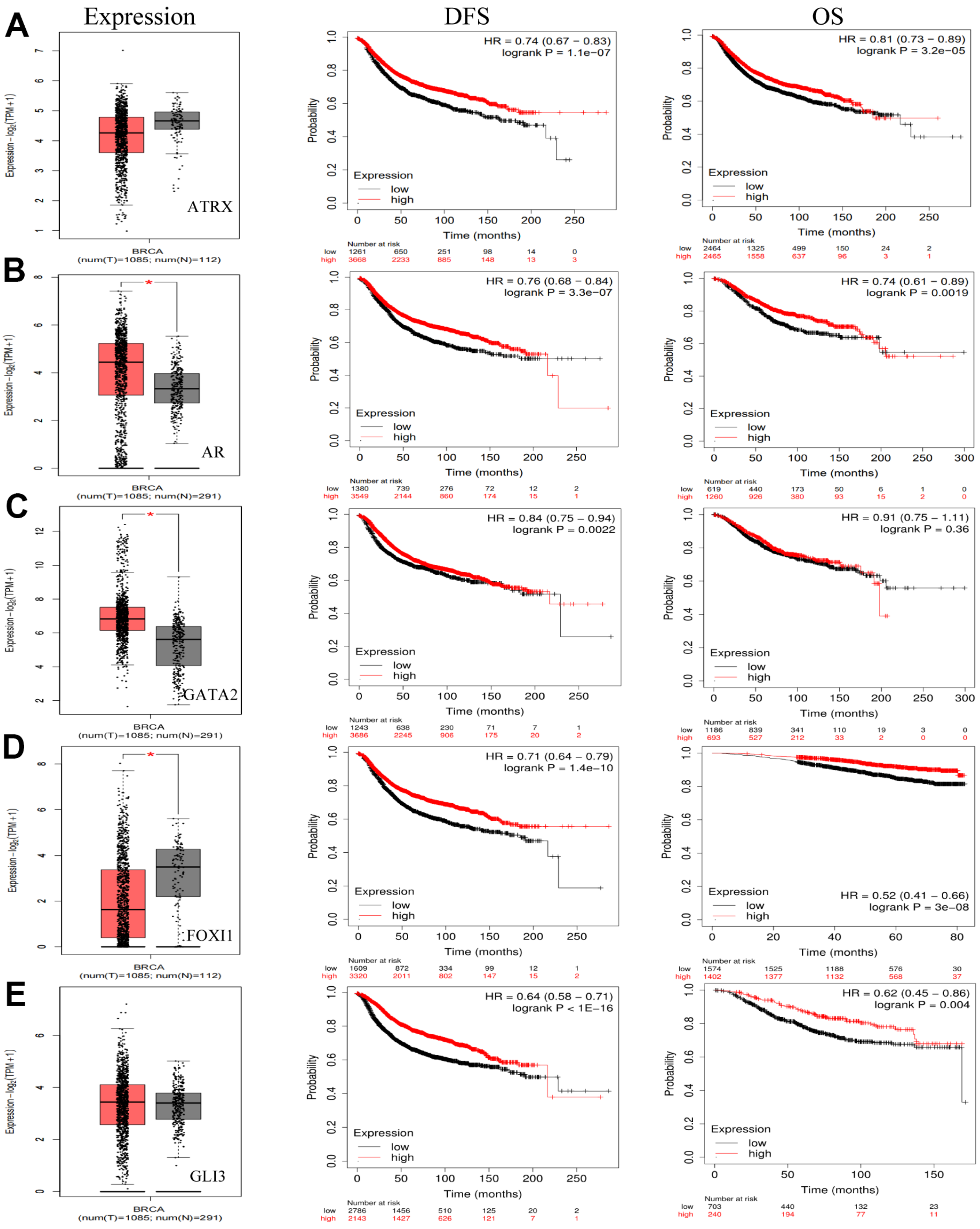

Supplementary Figure 2. Kaplan-Meier (KM) curves with positive results presented for BC in the TCGA based on the expression of ATRX, AR, GATA2, FOXI1, GLI3. KM curves analysis for the DFS and OS of BC patients based on the expression of ATRX (**A**), AR (**B**), GATA2 (**C**), FOXI1 (**D**), GLI3(**E**) in TCGA.

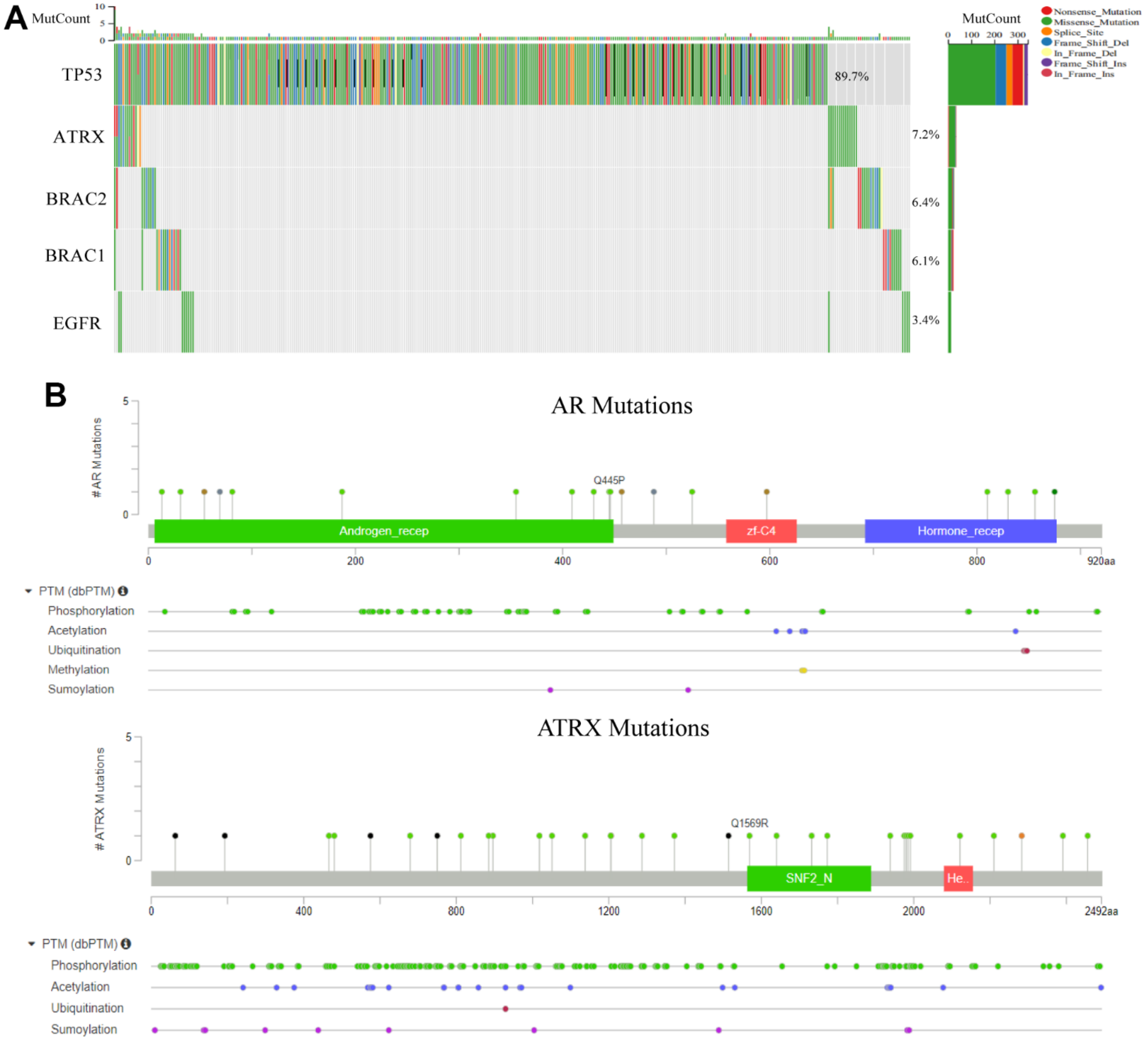

Supplementary Figure 3. Summary of mutation profiling in the BC samples from TCGA. (**A**) Landscape of mutation profiles in the BC samples from TCGA. Mutation information of each gene in each sample is shown in the waterfall plot. (**B**) Potential interaction mechanism of AR and ATRX by analyses of the landscape of the multiple modification sites.

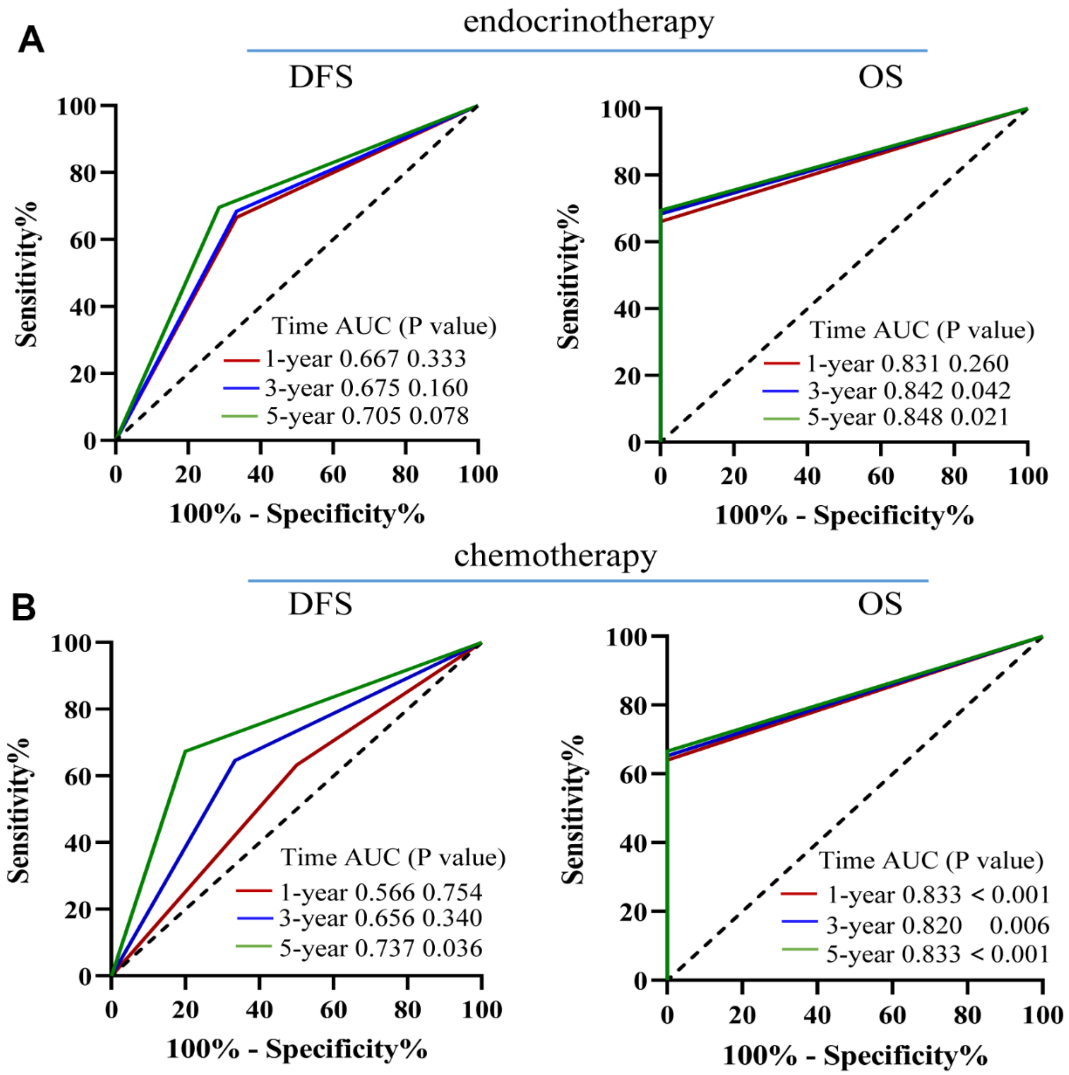

Supplementary Figure 4. Time-dependent receiver operating characteristics (ROC) analysis were used to evaluate the predictive ability of ARTX in the TMA data. (**A**) ROC curve analysis based on ATRX for predicting the 1-,3-,5-year DFS or 1-,3-,5-year OS of the HER2-/HR+ BC patients who underwent endocrinotherapy. (**B**) ROC curve analysis based on ATRX for predicting the 1-,3-,5-year DFS or 1-,3-,5-year OS of the HER2-/HR+ BC patients who underwent chemotherapy.
