# Supplementary material for: ATRX is a predictive marker for endocrinotherapy and chemotherapy resistance in HER2-/HR+ breast cancer through the regulation of the AR, GLI3 and GATA2 transcriptional network
Source: Aging (Albany NY). 2023 Dec 20;15(24):14996–5024. doi: 10.18632/aging.205327 (PMC10781474; doi:10.18632/aging.205327)
Supplement: Supplementary Tables [file aging-15-205327-s002.pdf]

SUPPLEMENTARY TABLES

Supplementary Table 1. GEO database information.

GEO database	Platforms	Number of samples	Description	Data type
GSE24460	GPL571	2	MCF-7(control)	Expression profiling by array
		2	MCF-7(Doxorubicin Resistance)	
GSE22513	GPL570	8	pCR breast biopsy(paclitaxel)	
		20	non-pCR breast biopsy(paclitaxel)	
GSE8562	GPL96	3	MCF7(control)	
		3	MCF7/XBP1(Estrogen Resistance)	

pCR, pathological complete response.

Supplementary Table 2. The number of DEGs in each module of WGCNA.

Modules	Number of DEGs
black	90
blue	159
brown	286
cyan	43
greenyellow	120
grey	41
magenta	63
midnightblue	40
pink	67
red	119
salmon	47
tan	52
turquoise	182
yellow	144
